# Metabolomic biomarkers correlating with hepatic lipidosis in dairy cows

**DOI:** 10.1186/1746-6148-10-122

**Published:** 2014-06-02

**Authors:** Sandro Imhasly, Hanspeter Naegeli, Sven Baumann, Martin von Bergen, Andreas Luch, Harald Jungnickel, Sarah Potratz, Christian Gerspach

**Affiliations:** 1 University of Zürich-Vetsuisse, Institute of Pharmacology and Toxicology, Zürich CH-8057, Switzerland; 2 Department of Metabolomics, Helmholtz Centre for Environmental Research – UFZ, Leipzig D-04318, Germany; 3 Department of Proteomics, Helmholtz Centre for Environmental Research –UFZ, Leipzig D-04318, Germany; 4 Department of Biotechnology, Chemistry and Environmental Engineering, Aalborg University, Aalborg DK-9100, Denmark; 5 Department of Product Safety, Federal Institute of Risk Assessment, Berlin D-10589, Germany; 6 Department of Farm Animals, University of Zürich, Zurich CH-8057, Switzerland

**Keywords:** Biomarker, Fatty liver, Lipidosis, Metabolomics

## Abstract

**Background:**

Hepatic lipidosis or fatty liver disease is a major metabolic disorder of high-producing dairy cows that compromises animal performance and, hence, causes heavy economic losses worldwide. This syndrome, occurring during the critical transition from gestation to early lactation, leads to an impaired health status, decreased milk yield, reduced fertility and shortened lifetime. Because the prevailing clinical chemistry parameters indicate advanced liver damage independently of the underlying disease, currently, hepatic lipidosis can only be ascertained by liver biopsy. We hypothesized that the condition of fatty liver disease may be accompanied by an altered profile of endogenous metabolites in the blood of affected animals.

**Results:**

To identify potential small-molecule biomarkers as a novel diagnostic alternative, the serum samples of diseased dairy cows were subjected to a targeted metabolomics screen by triple quadrupole mass spectrometry. A subsequent multivariate test involving principal component and linear discriminant analyses yielded 29 metabolites (amino acids, phosphatidylcholines and sphingomyelines) that, in conjunction, were able to distinguish between dairy cows with no hepatic lipidosis and those displaying different stages of the disorder.

**Conclusions:**

This proof-of-concept study indicates that metabolomic profiles, including both amino acids and lipids, distinguish hepatic lipidosis from other peripartal disorders and, hence, provide a promising new tool for the diagnosis of hepatic lipidosis. By generating insights into the molecular pathogenesis of hepatic lipidosis, metabolomics studies may also facilitate the prevention of this syndrome.

## Background

Hepatic lipidosis (also known as “fatty liver disease” or “fat cow syndrome”) is a common production problem of dairy cows occurring during the critical physiologic transition from pregnancy to lactation [1-3]. During the last decades, dairy cows have undergone an intense genetic selection to increase the milk yield, thereby reaching an enhanced performance level where the excessive demand for nutrients results in a severe energetic deficit at the onset of lactation [4-6]. A major adjustment to counteract this metabolic imbalance is the rapid mobilization of fat depots, thus providing non-esterified fatty acids as an energy source. Hepatic lipidosis develops when, during early lactation, the hepatic uptake of these non-esterified fatty acids and storage in the form of triacylglycerols exceeds their elimination [2,7]. The rate of triacylglycerol production in the liver tissue of ruminants is similar to that found in other species [8]. However, besides their use for energy production through mitochondrial breakdown by oxidation, triacylglycerols are released from hepatocytes as part of lipoproteins, whereby very low-density lipoproteins (VLDL) constitute the largest proportion. In ruminants, the secretion of VLDL from the liver is very limited compared with other species such that the resulting storage of excess lipids in hepatocytes leads to liver damage and depressed liver functions [8,9].

Hepatic lipidosis in cows is associated with ketosis, anorexia, reduced rumen motility, displaced abomasum, weight loss, predisposition to infections and diminished fertility [10,11], although the disease also occurs in a subclinical form affecting milk production and the long-term profitability of dairy farms. Indeed, previous reports suggest that this syndrome is a substantial problem for up to 50% of high-yielding cows [12,13] and, hence, hepatic lipidosis is recognized as a serious herd problem and animal welfare concern. Unfortunately, the diagnosis of hepatic lipidosis can only be confirmed by taking biopsies to determine the hepatic lipid content [8]. Biochemical abnormalities like increased liver enzymes and bilirubin concentrations in plasma correlate with advanced tissue damage but are not specific for hepatic lipidosis [14]. Thus, in view of the lack of a practically useful diagnostic tool, we explored an alternative strategy by screening for small-molecule biomarkers. The purpose of this study was to identify serum biomarkers that distinguish cows with hepatic lipidosis from those affected by other peripartal disorders.

## Methods

### Animal samples

Blood samples were taken from the jugular vein of Holstein-Friesian and Red-Holstein cows that contracted hepatic lipidosis or other diseases that typically occur during the peripartal period, including displaced abomasum, retained placenta or mastitis (Table 1). Plasma for clinical chemistry was prepared by supplementing blood samples with either heparin or EDTA. For metabolomic analyses, serum was collected and stored at -80°C. The diagnosis of hepatic lipidosis was obtained by liver biopsy and histologic inspection. The stage of disease was classified as described [15] according to the different extension of lipid deposition across the morphologic liver zones (periportal, transition zone and pericentral): group 1 (no lipid deposition), group 2 (only one zone affected), group 3 (all three zones affected), group 4 (Kupffer cells affected in addition to all three zones). Ethics approval was not requested and not needed because samples were taken from diseased animals during routine diagnostic investigations when hepatic lipidosis was suspected in peripartal dairy cows.

**Table 1:** Periparturient dairy cows of the study, their disease, mean age (years) and lactation stage (weeks postpartum)

**Group**	**N**	**Diseases (number of affected animals)**	**Age years ± S.D.**	**Lactation weeks ± S.D.**
1	6	Displaced abomasum (3), bronchopneumonia (2), ileus (1)	7.2 ± 1.8	1.8 ± 0.7
2	10	Low grade hepatic lipidosis (10) and, in addition, displaced abomasum (3), bronchopneumonia (1), retained placenta (1), mastitis (1)	5.1 ± 1.7	2.9 ± 2.1
3	7	Medium grade hepatic lipidosis (7) and, in addition, displaced abomasum (2) and bronchopneumonia (1)	5.2 ± 2.4	2.3 ± 0.7
4	5	Severe hepatic lipidosis (5) and, in addition, displaced abomasum (2) and bronchopneumonia (1)	5.5 ± 0.8	4.2 ± 1.3

The 28 animals were from 27 different farms, i.e., only two cows of group 3 came from the same farm.

### Clinical chemistry

Biochemical parameters were determined in plasma using the Cobas Integra 800 instrument (Roche Diagnostic, Rotkreuz, Switzerland). Aspartate aminotransferase (ASAT) was measured according to recommendations of the International Federation of Clinical Chemistry [16] with intra-assay and inter-assay coefficients of variance (CV) of 1.4% and 1.7%, respectively; γ-glutamyl transferase (GGT) was measured by a colorimetric assay [17] with intra-assay and inter-assay CV of 1.8%; further colorimetric assays [18,19] were employed for the determination of glutamate dehydrogenase (GLDH) and sorbitol dehydrogenase (SDH) activities. For GLDH, the intra-assay and inter-assay CV were 0.8% and 1.2%, respectively; the intra-assay and inter-assay CV for SDH measurements were 3.9% and 3.2%. Bilirubin levels (intra-assay CV of 2.4%) were assessed by a diazo method [20]. Total protein (intra-assay CV and inter-assay CV of 6.6% and 11.5%) and fibrinogen levels (intra-assay CV of 11.1%) were measured by refractometry [21].

### Metabolite quantification

The Absolute-IDQ platform (Kit p150, Biocrates, Innsbruck, Austria) was employed for targeted metabolite profiling as described by the manufacturer. This platform detects a total of 163 metabolites, including 14 amino acids, 41 acylcarnitines (Cx:y), hydroxylacylcarnitines [C (OH) x:y] and dicarboxylacylcarnitines (Cx:y-DC), the sum of hexoses, 15 sphingomyelins (SMx:y) and sphingomyelin derivatives [SM (OH) x:y], as well as 15 lyso-phosphatidylcholines and 77 phosphatidylcholines (PC). The latter were further differentiated with respect to the presence of ester (“a”) and ether (“e”) bonds in the glycerol moiety, whereby two letters “aa” (=diacyl) and “ae” (=acyl-alkyl) indicate that two glycerol positions are bound to a fatty acid residue, while a single letter “a” (=acyl) indicates the presence of a single fatty acid residue. The lipid side chain composition is abbreviated with “Cx:y”, whereby “x” denotes the number of carbons in the side chain and “y” the number of double bonds. A detailed list of all analyzed metabolites is presented elsewhere [22].

The assay was performed on a double-filter 96-well plate containing stable isotope-labeled internal standards. All chemicals were from Sigma-Aldrich (Steinheim, Germany). Briefly, the serum samples (10 μl) were pipetted onto the upper filter spots of the 96-well plate and phenylisothiocyanate was added for derivatization of amino acids. Next, the samples were dried under a nitrogen stream, extracted with 5 mM ammonium acetate in methanol, centrifuged through the filter membranes and diluted with chromatographic solvent. Finally, the extracts were injected into the Agilent 1100 Series HPLC (operated by the Analyst 1.4.2 software) coupled to an API 4000 triple quadrupole mass spectrometer (ABSciex) through electrospray ionization. A standard flow injection with two 20-μl aliquots (one for the positive and one for the negative ion mode) was applied to all measurements. Quantification was achieved by multiple reaction monitoring (MRM) detection using the MetIQTM software package, which is an integral part of the AbsoluteIDQ kit. This method is in conformity with the proof of reproducibility outlined in the Guidance for Industry–Bioanalytical Method Validations issued by the FDA [23]. The analytical variability, in terms of intra-assay coefficient of variance (CV), was 7.3%. For statistical analyses, only metabolites were chosen for which all values exceeded the detection limit, thus restricting the profile to a total of 80 metabolites (5 amino acids, 62 phosphatidylcholines, 8 sphingomyelins and 5 sphingomyelin derivatives). These metabolites were: glutamine, glycine, phenylalanine, proline, serine, PC aa C24:0, PC aa C26:0, PC aa C28:1, PC aa C30:0, PC aa C30:2, PC aa C32:0, PC aa C32:1, PC aa C32:2, PC aa C32:3, PC aa C34:1, PC aa C34:2, PC aa C34:3,PC aa C34:4, PC aa C36:1, PC aa C36:2, PC aa C36:3, PC aa C36:4, PC aa C36:5, PC aa C36:6, PC aa C38:3, PC aa C38:4, PC aa C38:5, PC aa C38:6, PC aa C40:2, PC aa C40:3, PC aa C40:4, PC aa C40:5, PC aa C40:6, PC aa C42:1, PC aa C42:2, PC ae C30:1, PC ae C30:2, PC ae C32:1, PC ae C32:2, PC ae C34:0, PC ae C34:1, PC ae C34:2, PC ae C34:3, PC ae C36:0, PC ae C36:1, PC ae C36:2, PC ae C36:3, PC ae C36:5, PC ae C38:1, PC ae C38:2, PC ae C38:3, PC ae C38:4, PC ae C38:5, PC ae C38:6, PC ae C40:2, PC ae C40:3, PC ae C40:5, PC ae C40:6, PC ae C42:2, PC ae C42:3, lysoPC a C16:0, lysoPC a C16:1, lysoPC a C18:0, lysoPC a C18:1, lysoPC a C26:0, lysoPC a C28:0, lysoPC a C28:1, SM C16:0, SM C16:1, SM C18:0, SM C18:1, SM C24:0, SM C24:1, SM C26:0, SM C26:1, SM (OH) C14:1, SM (OH) C16:1, SM (OH) C22:1, SM (OH) C22:2 and SM (OH) C24:1.

The mass spectrometry data of this study were deposited in the PRIDE database using the mzML format (accession number 1-20130722-115242).

### Statistics

A multivariate processing of metabolomics data was carried out using the statistical package SPSS + (version 12.0.2G). First, each sample was standardized to the mean of the control (set to 100%) and normalized using z-score values. Then, a MANOVA (multivariate analysis of variance) was used for compound selection [24,25]. All 29 variables that showed a significant group difference (p ≤ 0.006) were selected for a principal component analysis-linear discriminant function model. The principal component analysis (without rotation) was performed to achieve data reduction and the resulting factors were used for a post-hoc linear discriminant analysis as described elsewhere [26] for group separation. This linear discriminant analysis model resulted in three factors accounting for 100% of the observed variance in the system. The linear functions for these three discriminant factors were: F(x_1_) = 1.45 • VAR1 + 2.67 • VAR2 – 0.99 • VAR3 – 2.263 • VAR4 + 1.11 • VAR5 + 0.298 • VAR6 + 1.15 • VAR7 + 1.05 • VAR8 – 0.94 • VAR9 – 0.083 • VAR10 – 1.08 • VAR11; F(*x*_2_) = – 1.19 • VAR1 + 0.59 • VAR2 + 0.024 • VAR3 – 0.774 • VAR4 – 0.72 • VAR5 + 0.18 • VAR6 – 0.46 • VAR7 + 0.71 • VAR8 + 0.32 • VAR9 + 0.42 • VAR10 + 0.61 • VAR11; F(x_3_) = - 0.113 • VAR1 – 0.22 • VAR2 + 0.57 • VAR3 – 0.120 • VAR4 – 0.11 • VAR5 – 0.22 • VAR6 + 0.57 • VAR7 – 0.21 • VAR8 + 0.41 • VAR9 – 0.26 • VAR10 + 0.64 • VAR11. The discriminant function F(x_1_) separates the fatty liver group 3 from control animals (group 1) as well as from group 2. F(x_1_) also discriminates between groups 1 and 2. The discriminant function F(*x*_2_) separates controls (group 1) from group 4, whereas F(x_3_) discriminates between groups 2 and 4. A detailed description of the equations for VAR1 to VAR11 is included [see Additional file 1]. By this method, all samples were classified correctly in the corresponding hepatic lipidosis groups defined by histopathological findings. The performance of this discriminant model was subsequently verified by applying the “leave-one-out” cross-validation formalism [27,28].

## Results

A total of 28 diseased cows (from 27 different farms) were tested to identify metabolic biomarkers distinguishing hepatic lipidosis from other peripartal disorders. Following liver biopsy and histologic examination, the 28 early lactating cows were partitioned into 4 categories: group 1 (constituting the reference group of 6 animals displaying no hepatic lipidosis), group 2 (10 animals with low grade hepatic lipidosis), group 3 (7 animals with medium grade hepatic lipidosis) and group 4 (5 animals with severe hepatic lipidosis). The animals of group 1 were presented to the veterinary hospital because of displaced abomasum, bronchopneumonia or ileus. Some of these disorders, besides retained placenta or mastitis, were also encountered in the animals of groups 2–4 in addition to their different stages of hepatic lipidosis (Table 1).

### Clinical chemistry analysis

**Figure 1:**
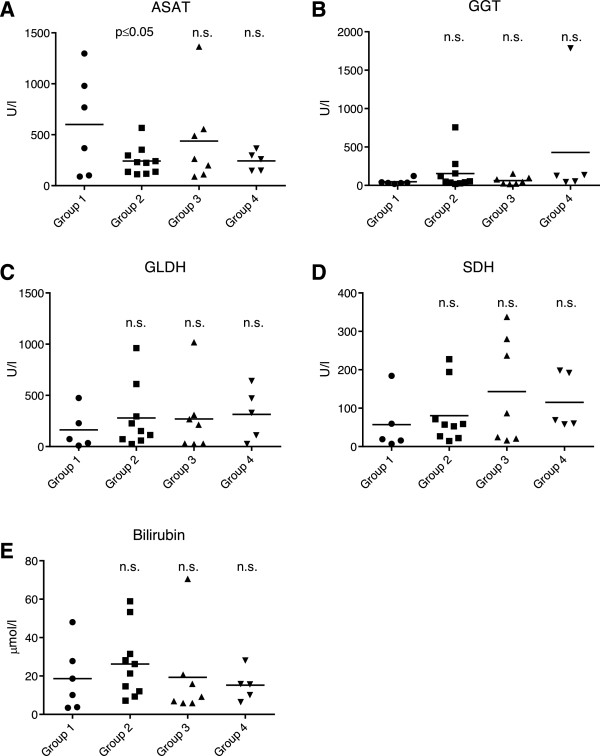
**Comparison of clinical chemistry parameters between the reference animals of group 1 (without hepatic lipidosis) and groups 2–4 (with progressive stages of hepatic lipidosis).** The parameters examined were **(A)** Aspartate aminotransferase (ASAT), **(B)** γ-glutamyl transferase (GGT), **(C)** glutamate dehydrogenase (GLDH), **(D)** Sorbitol dehydrogenase (SDH) and **(E)** Bilirubin. *P* ≤ 0.05 indicates a significant reduction of ASAT activity in the animals of group 2 relative to group 1; n.s., not significant.

**Figure 2:**
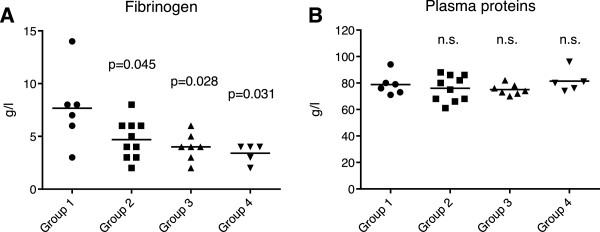
**Level of fibrinogen in plasma and total plasma protein concentration in the different groups of dairy cows (mean values of 5–10 animals). (A)** Plasma fibrinogen. **(B)** Total plasma protein. Group 1: no hepatic lipidosis; groups 2–4: progressive stages of hepatic lipidosis. The *P* values indicate significant differences with the reference group 1; n.s., not significant.

In dairy cows, the excess storage of triacylglycerols in the liver causes progressive hepatocyte damage and, consequently, membrane leakage that results in the increased release of liver enzymes and bile constituents into the blood [2,9]. However, clinical chemical parameters like aspartate aminotransferase (ASAT), γ-glutamyl transferase (GGT), glutamate dehydrogenase (GLDH), sorbitol dehydrogenase (SDH) and bilirubin levels in the blood, being non-specific biomarkers of organ injury, were already elevated in the reference group 1 of cows whose biopsies did not reveal typical features of hepatic lipidosis but that were admitted to the veterinary hospital for other disorders (Table 2). As a general trend, some of these conventional clinical chemistry values (GGT, GLDH and SDH) further increased from group 1 to group 4 (Figure 1), but without being able to discriminate between distinct disease etiologies. Conversely, we observed decreasing plasma fibrinogen concentrations correlating with the gradually enhanced severity of fatty liver disease in groups 2–4 relative to group 1 (Figure 2A), although the overall protein level remained in the normal range (Figure 2B). In summary, these selected clinical chemistry parameters fail to display specificity for the appearance of hepatic lipidosis and, hence, are not sufficient to confirm the diagnosis of this particular disease.

**Table 2:** Key clinical chemistry values in the blood of the reference group 1 (without hepatic lipidosis)

**Parameter**	**Normal range**	**Measured values in the reference animals (group 1)**		
		**Median**	**Mean**	**Standard error of the mean**
ASAT	57-103 U/l	568 U/l	600 U/l	202 U/l
GGT	13-32 U/l	34 U/l	48 U/l	16 U/l
GLDH	4-18.2 U/l	73 U/l	164 U/l	86 U/l
SDH	4-7.4 U/l	19 U/l	57 U/l	33 U/l
Total bilirubin	1.5-2.9 μmol/l	14.4 μmol/l	18.6 μmol/l	7.0 μmol/l

### Metabolomic analysis

**Figure 3:**
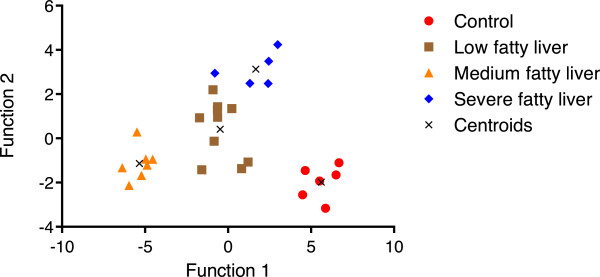
**Combined principal component and linear discriminant analysis.** This model reveals that 29 endogenous metabolites, in conjunction, distinguish dairy cows with different stages of hepatic lipidosis (groups 2–4) from reference animals (group 1) lacking the characteristic lipid deposition in their liver. The centroid, or center of mass, were computed from the coordinates of all points in each group. The linear functions for the two main discriminant factors [F (x_1_) and F (*x*_2_)] are laid down in the “Methods” section.

To expand the spectrum of biomarkers that accompany hepatic lipidosis in dairy cows, the AbsoluteIDQ system was applied in conjunction with triple quadrupole mass spectrometry to compare the serum metabolites of animals that did not show any lipid storage in liver cells (reference group 1) and the corresponding groups 2–4 displaying various stages of the syndrome. The AbsoluteIDQ metabolomic platform allows for the accurate identification and quantitative measurement of 163 endogenous metabolites from different compound classes, including amino acids, carbohydrates, carnitines, sphingolipids and phosphatidylcholines [29-31]. A multivariate statistical model revealed 29 metabolites, listed in Table 3, who could be used to separate the hepatic lipidosis groups 2 to 4 from the reference group 1. To graphically illustrate the clear separation between groups achieved by this set of metabolic endpoints, the data were subjected to principal component and linear discriminant analyses (Figure 3). Using the loading factors for linear discriminant function 1 outlined in the “Methods” section, we identified 6 phosphatidylcholines that contributed the most to the observed separation and could therefore be regarded as a promising predictive biomarkers of hepatic lipidosis: PC aa C30:2, PC aa C32:2, PC aa C36:3, PC aa C38:3, PC aa C36:4 and PC ae C36:2).

**Table 3:** List of metabolites with significant changes between the groups upon MANOVA (normalized mean values ± S.D.)

**Metabolite**	**Category**	**Group 1**	**Group 2**	**Group 3**	**Group 4**	***P *** **value**
Glutamine	Amino acids	100 ± 28.7	50.1 ± 24.3	49.6 ± 13.4	30.8 ± 5.9	≤ 0.0004
Glycine		100 ± 21.9	66.6 ± 23.4	52.0 ± 11.8	49.0 ± 9.3	≤ 0.0004
PC aa C30:2	Phosphatidyl-cholines	100 ± 15.7	87.7 ± 24.1	118.4 ± 32.9	139.0 ± 16.2	0.004
PC aa C32:2		100 ± 17.3	81.7 ± 38.4	121.0 ± 62.0	159.0 ± 39.7	0.002
PC aa C36:2		100 ± 15.0	66.2 ± 33.8	52.8 ± 13.9	40.8 ± 10.8	0.002
PC aa C36:3		100 ± 15.2	58.4 ± 20.7	50.5 ± 14.4	50.0 ± 13.0	≤ 0.0004
PC aa C36:4		100 ± 11.5	60.8 ± 21.7	50.2 ± 13.4	51.6 ± 14.4	≤ 0.0004
PC aa C38:3		100 ± 14.5	58.3 ± 20.7	49.1 ± 14.7	45.9 ± 10.3	≤ 0.0004
PC aa C38:4		100 ± 11.7	70.7 ± 22.2	55.3 ± 21.0	59.5 ± 14.9	0.002
PC aa C38:6		100 ± 15.5	70.1 ± 16.0	79.1 ± 12.4	78.5 ± 12.7	0.006
PC aa C40:2		100 ± 17.7	70.6 ± 39.5	51.0 ± 21.4	34.1 ± 12.0	0.004
PC aa C40:3		100 ± 18.3	45.0 ± 18.4	40.1 ± 12.6	32.3 ± 6.9	≤ 0.0004
PC aa C40:4		100 ± 10.6	68.0 ± 26.6	55.1 ± 17.5	49.2 ± 10.2	0.001
PC aa C42:2		100 ± 11.2	76.4 ± 35.9	64.7 ± 25.9	36.6 ± 9.4	0.005
PC ae C34:1		100 ± 12.7	71.4 ± 23.9	62.0 ± 10.3	58.1 ± 10.6	0.001
PC ae C36:2		100 ± 15.8	63.7 ± 24.5	58.6 ± 10.6	55.7 ± 12.1	0.001
PC ae C36:3		100 ± 17.8	61.3 ± 14.3	58.3 ± 12.8	60.8 ± 8.8	≤ 0.0004
PC ae C38:2		100 ± 14.8	73.9 ± 31.8	54.3 ± 16.6	42.6 ± 7.5	0.001
PC ae C38:3		100 ± 11.7	56.9 ± 18.2	50.0 ± 15.8	41.3 ± 8.0	≤ 0.0004
PC ae C38:4		100 ± 17.8	67.4 ± 14.1	68.8 ± 17.1	63.7 ± 14.2	0.002
PC ae C40:2		100 ± 7.6	62.5 ± 17.4	62.9 ± 9.9	57.1 ± 12.6	≤ 0.0004
PC ae C40:3		100 ± 11.5	61.7 ± 13.2	65.1 ± 10.3	75.2 ± 10.2	≤ 0.0004
SM C18:0	Sphingomyelins	100 ± 22.4	67.2 ± 34	49.2 ± 48.6	14.2 ± 20.5	0.003
SM C18:1		100 ± 21.9	66.9 ± 33.3	53.5 ± 20.2	34.0 ± 10.2	0.002
SM C24:1		100 ± 13.7	52.2 ± 16.5	62.3 ± 15.5	47.8 ± 9.7	≤ 0.0004
SM C26:1		100 ± 14.8	45.9 ± 16.3	57.8 ± 12.9	42.2 ± 7.9	≤ 0.0004
SM (OH) C22:1	Hydroxy-sphingomyelins	100 ± 13.0	52.1 ± 18.1	68.8 ± 16.6	67.7 ± 12.8	≤ 0.0004
SM (OH) C22:2		100 ± 10.6	48.0 ± 15.4	56.5 ± 15.9	55.6 ± 11.9	≤ 0.0004
SM (OH) C24:1		100 ± 6.0	56.3 ± 21.8	74.2 ± 15.2	58.0 ± 13.9	≤ 0.0004

Group 1, without hepatic lipidosis; groups 2–4, progressive stages of hepatic lipidosis.

**Figure 4:**
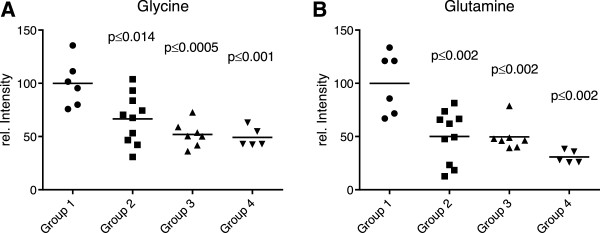
**Typical examples of metabolites whose concentrations decreased with progressing hepatic lipidosis.** Group 1: no hepatic lipidosis; groups 2–4: different stages of hepatic lipidosis (mean values of 5–10 animals). **(A)** Level of glycine in the serum of dairy cows. **(B)** Level of glutamine in the serum of dairy cows. The *P* values indicate significant differences with the reference group 1.

**Figure 5:**
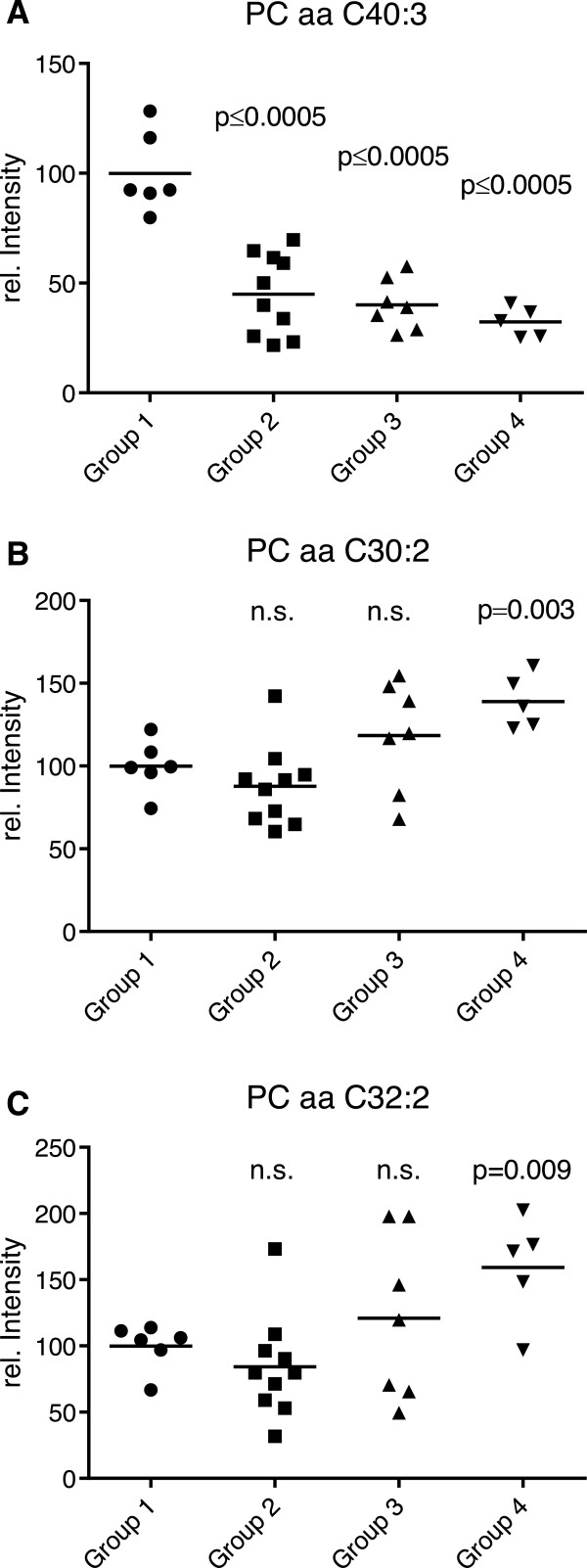
**Serum concentration of diacyl-phosphatidylcholines (mean values of 5–10 animals). (A)** Example of metabolite (PC aa C40:3) whose concentration decreased with progressing hepatic lipidosis. Group 1: no hepatic lipidosis; groups 2–4: different stages of hepatic lipidosis. **(B)** and **(C)** Diacyl-phosphatidylcholines whose serum concentrations increased with progressing hepatic lipidosis. The *P* values indicate significant differences with the reference group 1; n.s., not significant.

Two of the 29 discriminating metabolites turned out to be the amino acids glycine (Figure 4A) and glutamine (Figure 4B) with diminished blood levels in the diseased animals of groups 2–4 relative to group 1. However, the majority of metabolites able to discriminate between the reference group 1 and the different stages of fatty liver disease were phosphatidylcholines (PC), i.e. the diacyl-phosphatidylcholines PC aa C30:2, PC aa C32:2, PC aa C36:2, PC aa C36:3, PC aa C36:4, PC aa C38:3, PC aa C38:4, PC aa C38:6, PC aa C40:2, PC aa C40:3, PC aa C40:4 and PC aa C42:2, as well as the acyl-alkyl-phosphatidylcholines PC ae C34:1, PC ae C36:2, PC ae C36:3, PC ae C38:2, PC ae C38:3, PC ae C38:4, PC ae C40:2 and PC ae C40:3 (Table 3). Additional discriminating components in the tested sera included the sphingomyelins SM C18:0 (the only saturated lipid), SM C18:1, SM C24:1 and SM C26:1 as well as the hydroxy-sphingomyelines SM(OH) C22:1, SM (OH) C22:2 and SM (OH) C24:1. As exemplified by PC aa C40:3 in Figure 5A, generally these metabolites showed lower levels in cows with fatty liver disease (groups 2–4) than in the reference animals of group 1. Exceptions were the diacyl-phosphatidylcholines PC aa C30:2 and PC aa C32:2, for which higher levels could be measured in cows with hepatic lipidosis compared to the reference animals (Figures 5B and 5C). All lyso-phosphatidylcholines and the sum of hexoses did not show any significant difference between the four groups of early lactating cows.

## Discussion

All cows of this study displayed increased serum levels of enzymes and bilirubin that are generally indicative of liver damage or injuries to other tissues like cardiac or skeletal muscle [21]. Therefore, these canonical clinical chemistry values were unable to distinguish between hepatic lipidosis and other peripartal disorders. A novel observation of this study is the reduction of the fibrinogen serum conten in periparturient cows suffering from hepatic lipidosis. As this fibrin precursor is produced in the liver, its lower level may represent a direct consequence of hepatocyte dysfunction due to excessive lipid deposition, but may also result from coagulopathy linked to liver disease [32]. In fact, hyperfibrinolysis leading to hypofibrinogenemia is known to arise from a poor hepatic clearance of tissue plasminogen activator or from reduced hepatic production of fibrinolysis inhibitors. However, there was no clinically apparent coagulopathy in the cows of the current study.

In view of the lack of specificity of canonical clinical chemistry values indicative of hepatic lipidosis, including the aforementioned fibrinogen levels, the purpose of our pilot study was to establish a targeted metabolomics platform to discover correlations between this disorder and molecular changes detectable in blood samples of periparturient dairy cows. Instead of comparing groups of healthy and diseased animals, we employed a more practical situation encountered in the veterinary hospital environment, where hepatic lipidosis has to be differentiated from other peripartal diseases. As a consequence, the reference group in our study consisted of animals whose clinical chemistry parameters revealed considerable injuries to the liver or other tissue damages (Figure 1). Nevertheless, a targeted metabolomics approach led to the identification of an endogenous molecular profile that appears to be specific for hepatic lipidosis, thus distinguishing this disease from other peripartal problems. Two of the 29 discriminating metabolites were the amino acids glycine and glutamine that have already been linked to metabolic disorders and chronic inflammatory conditions [33-37], which are both key hallmarks of hepatic lipidosis. From the discriminant function coefficients of the variables we found, however, that the following six phosphatidylcholines contributed the most to the observed separation and could therefore be regarded as a potential set of predictive biomarkers of hepatic lipidosis: PC aa C30:2 (Figure 5B), PC aa C32:2 (Figure 5C), PC aa C36:3, PC aa C38:3, PC aa C36:4 and PC ae C36:2. Although changes of phosphatidylcholine and sphingomyelin blood levels have previously been associated with metabolic disorders [38] or chronic liver diseases [39], the possible link of the listed phosphatidylcholines with hepatic lipidosis is intriguing. Because they constitute an important precursor for the synthesis of hepatic triacylglycerols [40], phosphatidylcholines may be reduced in peripartal dairy cows as a direct consequence of an enhanced triacylglycerol production [2,7,8]. Conversely, hepatic phosphatidylcholines are required for the assembly and secretion of VLDL implying that a reduced phosphatidylcholine content may aggravate the accumulation of triacylglycerols in the liver by limiting their export from hepatocytes [41]. In our study, the observed changes of lipid levels involve an increase of those phosphatidylcholines that carry relatively short fatty acid moieties (PC aa C30:2 and PC aa C32:2), whereas the quantity of phosphatidylcholines containing larger fatty acid components (≥34 carbons) was reduced. This shift in phosphatidylcholine composition suggests a possible prophylactic or therapeutic approach based on the modulation of phosphatidylcholine biosynthesis by appropriate feed supplementation [42]. In any case, the observed shift in phosphatidylcholine distribution is a novel finding that needs to be further investigated mechanistically to understand if it has a causal relationship with the disease or rather constitutes a consequence thereof.

## Conclusion

New biomarkers of hepatic lipidosis are urgently needed to facilitate diagnostic procedures, identify distinct stages of the disease, monitor the response to treatment regimens and allow for the design of prevention strategies. An important new paradigm in biomarker discovery research is to consider entire sets of molecular changes, instead of single parameters, that correlate with a particular disease [43]. In the present study, we have exploited the fact that serum metabolite concentrations provide a direct readout of disturbed biochemical pathways [29]. This approach led us to employ multivariate statistics, based on 29 key metabolites, to recognize deranged metabolic patterns that correlate specifically with distinct stages of hepatic lipidosis in dairy cows, thus distinguishing this disease from other peripartal disorders. Future studies with larger animal groups are needed to confirm the findings of this study, to validate the newly identified metabolic profile and explore its clinical application to the diagnosis and treatment of diseased animals.

## Competing interests

The authors declare that they have no competing interests.

## Authors’ contributions

SI was involved in the metabolomics measurements and statistical analysis, HN, CG and AL initiated and organized the study, SB, MvB, HJ and SP participated in the metabolomics analysis and statistical evaluation, HJ and HN wrote the manuscript that was subsequently reviewed by all co-authors. All authors read and approved the final manuscript.

## Supplementary Material

Additional file 1 Equations for VAR1 to VAR11. Click here for file
