# Supplementary material for: Metabolomic biomarkers correlating with hepatic lipidosis in dairy cows
Source: BMC Vet Res. 2014 Jun 2;10:122. doi: 10.1186/1746-6148-10-122 (PMC4048253; doi:10.1186/1746-6148-10-122)
Supplement: Additional file 1 — Equations for VAR1 to VAR11. [file 1746-6148-10-122-S1.doc]

**Additional information**

**Equations for variables VAR1 to VAR11 (see main text for abbreviation of the different metabolites):**

VAR1 = 0.039 • Gln + 0.037 • Gly + 0.042 • PC aa C36:3 + 0.042 • PC aa C36:4 + 0.042 • PC aa C38:3 + 0.041 • PC aa C40:3 + 0.038 • PC ae C36:3 + 0.041 • PC ae C38:3 + 0.41 • PC ae C40:2 + 0.031 • PC ae C40:3 + 0.30 • SM (OH) C22:1 + 0.036 • SM(OH) C22:2 + 0.035 • SM(OH) C24:1 + 0.039 • SM C24:1 + 0.037 • SM C26:1 + 0.039 • PC aa C40:4 + 0.042 • PC ae C34:1 + 0.042 • PC ae C36:2 + 0.040 • PC ae C38:2 – 0.008 • PC aa C32:2 + 0.041 • PC aa C36:2 + 0.038 • PC aa C38:4 + 0.039 • PC ae C38:4 + 0.039 • SM C18:1 + 0.030 • SM C18:0 – 0.009 • PC aa C30:2 + 0.037 • PC aa C40:2 + 0.034 • PC aa C42:2 + 0.30 • PC aa C38:6

VAR2 = 0.011 • Gln – 0.041 • Gly + 0.023 • PC aa C36:3 + 0.013 • PC aa C36:4 + 0.006 • PC aa C38:3 + 0.021 • PC aa C40:3 + 0.070 • PC ae C36:3 + 0.015 • PC ae C38:3 + 0.43 • PC ae C40:2 + 0.114 • PC ae C40:3 + 0.132 • SM (OH) C22:1 + 0.105 • SM(OH) C22:2 + 0.087 • SM(OH) C24:1 + 0.078 • SM C24:1 + 0.088 • SM C26:1 – 0.047 • PC aa C40:4 – 0.008 • PC ae C34:1 + 0.020 • PC ae C36:2 – 0.076 • PC ae C38:2 + 0.190 • PC aa C32:2 – 0.045 • PC aa C36:2 – 0.011 • PC aa C38:4 + 0.035 • PC ae C38:4 – 0.066 • SM C18:1 – 0.093 • SM C18:0 + 0.193 • PC aa C30:2 – 0.080 • PC aa C40:2 – 0.068 • PC aa C42:2 + 0.115 • PC aa C38:6

VAR3 = 0.046 • Gln + 0.048 • Gly – 0.078 • PC aa C36:3 – 0.060 • PC aa C36:4 – 0.093 • PC aa C38:3 – 0.137 • PC aa C40:3 – 0.058 • PC ae C36:3 – 0.154 • PC ae C38:3 + 0.019 • PC ae C40:2 – 0.131 • PC ae C40:3 – 0.107 • SM (OH) C22:1 – 0.191 • SM(OH) C22:2 + 0.022 • SM(OH) C24:1 + 0.032 • SM C24:1 – 0.138 • SM C26:1 + 0.001 • PC aa C40:4 + 0.136 • PC ae C34:1 + 0.078 • PC ae C36:2 + 0.086 • PC ae C38:2 + 0.399 • PC aa C32:2 + 0.132 • PC aa C36:2 - 0.129 • PC aa C38:4 – 0.108 • PC ae C38:4 – 0.004 • SM C18:1 – 0.282 • SM C18:0 + 0.303 • PC aa C30:2 + 0.221 • PC aa C40:2 + 0.312 • PC aa C42:2 + 0.152 • PC aa C38:6

VAR4 = – 0.695 • Gln – 0.890 • Gly + 0.085 • PC aa C36:3 + 0.078 • PC aa C36:4 + 0.031 • PC aa C38:3 – 0.237 • PC aa C40:3 + 0.010 • PC ae C36:3 + 0.145 • PC ae C38:3 + 0.068 • PC ae C40:2 – 0.465 • PC ae C40:3 + 0.254 • SM (OH) C22:1 + 0.132 • SM(OH) C22:2 + 0.136 • SM(OH) C24:1 – 0.057 • SM C24:1 – 0.086 • SM C26:1 + 0.114 • PC aa C40:4 + 0.105 • PC ae C34:1 + 0.127 • PC ae C36:2 + 0.050 • PC ae C38:2 – 0.059 • PC aa C32:2 + 0.046 • PC aa C36:2 + 0.260 • PC aa C38:4 – 0.121 • PC ae C38:4 + 0.097 • SM C18:1 + 0.361 • SM C18:0 + 0.247 • PC aa C30:2 + 0.185 • PC aa C40:2 – 0.003 • PC aa C42:2 + 0.255 • PC aa C38:6

VAR5 = 0.114 • Gln + 0.191 • Gly + 0.025 • PC aa C36:3 – 0.004 • PC aa C36:4 + 0.051 • PC aa C38:3 – 0.046 • PC aa C40:3 + 0.197 • PC ae C36:3 – 0.132 • PC ae C38:3 – 0.309 • PC ae C40:2 – 0.726 • PC ae C40:3 – 0.006 • SM (OH) C22:1 – 0.020 • SM(OH) C22:2 + 0.003 • SM(OH) C24:1 + 0.084 • SM C24:1 – 0.080 • SM C26:1 – 0.312 • PC aa C40:4 – 0.068 • PC ae C34:1 – 0.027 • PC ae C36:2 – 0.035 • PC ae C38:2 + 0.405 • PC aa C32:2 + 0.020 • PC aa C36:2 + 0.242 • PC aa C38:4 + 1.120 • PC ae C38:4 + 0.273 • SM C18:1 + 0.469 • SM C18:0 + 0.545 • PC aa C30:2 – 0.133 • PC aa C40:2 – 0.058 • PC aa C42:2 – 0.831 • PC aa C38:6

VAR6 = – 0.357 • Gln + 0.495 • Gly – 0.244 • PC aa C36:3 + 0.061 • PC aa C36:4 – 0.101 • PC aa C38:3 – 0.091 • PC aa C40:3 – 0.591 • PC ae C36:3 – 0.611 • PC ae C38:3 – 0.270 • PC ae C40:2 + 0.647 • PC ae C40:3 + 0.612 • SM (OH) C22:1 + 0.003 • SM(OH) C22:2 + 0.253 • SM(OH) C24:1 – 0.304 • SM C24:1 – 0.368 • SM C26:1 + 0.745 • PC aa C40:4 – 0.063 • PC ae C34:1 – 0.232 • PC ae C36:2 – 0.192 • PC ae C38:2 – 0.174 • PC aa C32:2 – 0.106 • PC aa C36:2 + 0.170 • PC aa C38:4 + 0.199 • PC ae C38:4 + 0.436 • SM C18:1 + 0.139 • SM C18:0 + 0.486 • PC aa C30:2 + 0.190 • PC aa C40:2 – 0.411 • PC aa C42:2 + 0.066 • PC aa C38:6

VAR7 = – 1.021 • Gln + 0.705 • Gly – 0.239 • PC aa C36:3 + 0.219 • PC aa C36:4 + 0.562 • PC aa C38:3 + 0.826 • PC aa C40:3 + 1.024 • PC ae C36:3 – 0.427 • PC ae C38:3 + 0.288 • PC ae C40:2 – 0.171 • PC ae C40:3 + 0.676 • SM (OH) C22:1 + 0.474 • SM(OH) C22:2 – 1.319 • SM(OH) C24:1 + 0.176 • SM C24:1 – 0.020 • SM C26:1 – 0.596 • PC aa C40:4 – 0.031 • PC ae C34:1 – 0.683 • PC ae C36:2 – 0.647 • PC ae C38:2 + 0.050 • PC aa C32:2 – 0.506 • PC aa C36:2 – 0.415 • PC aa C38:4 – 0.167 • PC ae C38:4 – 0.389 • SM C18:1 – 0.005 • SM C18:0 + 0.152 • PC aa C30:2 + 0.684 • PC aa C40:2 + 0.429 • PC aa C42:2 – 0.014 • PC aa C38:6

VAR8 = 0.167 • Gln – 0.128 • Gly – 0.328 • PC aa C36:3 + 0.108 • PC aa C36:4 – 1.161 • PC aa C38:3 + 0.035 • PC aa C40:3 – 0.013 • PC ae C36:3 – 0.263 • PC ae C38:3 + 1.562 • PC ae C40:2 – 0.370 • PC ae C40:3 – 0.299 • SM (OH) C22:1 + 0.447 • SM(OH) C22:2 + 0.112 • SM(OH) C24:1 + 0.058 • SM C24:1 – 0.479 • SM C26:1 + 1.349 • PC aa C40:4 + 0.209 • PC ae C34:1 – 0.053 • PC ae C36:2 – 0.121 • PC ae C38:2 + 0.592 • PC aa C32:2 – 0.145 • PC aa C36:2 – 0.711 • PC aa C38:4 + 1.002 • PC ae C38:4 – 1.728 • SM C18:1 + 0.640 • SM C18:0 – 0.586 • PC aa C30:2 – 0.816 • PC aa C40:2 – 0.530 • PC aa C42:2 + 0.216 • PC aa C38:6

VAR9 = – 1.628 • Gln + 1.637 • Gly + 0.436 • PC aa C36:3 – 1.827 • PC aa C36:4 – 0.494 • PC aa C38:3 – 0.932 • PC aa C40:3 + 0.487 • PC ae C36:3 + 4.216 • PC ae C38:3 – 0.301 • PC ae C40:2 – 0.080 • PC ae C40:3 – 0.278 • SM (OH) C22:1 + 0.612 • SM(OH) C22:2 + 0.222 • SM(OH) C24:1 – 0.591 • SM C24:1 – 0.067 • SM C26:1 + 0.997 • PC aa C40:4 – 2.169 • PC ae C34:1 – 0.554 • PC ae C36:2 + 1.568 • PC ae C38:2 + 0.282 • PC aa C32:2 + 0.186 • PC aa C36:2 – 1.279 • PC aa C38:4 + 0.634 • PC ae C38:4 + 0.124 • SM C18:1 – 1.133 • SM C18:0 + 0.759 • PC aa C30:2 + 0.777 • PC aa C40:2 – 0.848 • PC aa C42:2 – 0.149 • PC aa C38:6

VAR10 = – 3.605 • Gln + 1.801 • Gly – 1.177 • PC aa C36:3 + 2.571 • PC aa C36:4 + 0.045 • PC aa C38:3 – 2.049 • PC aa C40:3 – 0.598 • PC ae C36:3 + 1.801 • PC ae C38:3 + 1.496 • PC ae C40:2 + 0.426 • PC ae C40:3 – 1.291 • SM (OH) C22:1 – 4.051 • SM(OH) C22:2 – 0.437 • SM(OH) C24:1 + 1.199 • SM C24:1 + 3.403 • SM C26:1 + 0.773 • PC aa C40:4 + 2.225 • PC ae C34:1 – 3.358 • PC ae C36:2 – 0.127 • PC ae C38:2 + 2.038 • PC aa C32:2 – 2.004 • PC aa C36:2 + 0.416 • PC aa C38:4 + 0.522 • PC ae C38:4 + 2.633 • SM C18:1 + 0.965 • SM C18:0 – 1.247 • PC aa C30:2 – 2.999 • PC aa C40:2 + 1.766 • PC aa C42:2 + 1.222 • PC aa C38:6

VAR11 = 1.085 • Gln – 1.378 • Gly – 7.162 • PC aa C36:3 – 2.398 • PC aa C36:4 – 3.430 • PC aa C38:3 – 0.279 • PC aa C40:3 + 1.574 • PC ae C36:3 + 0.674 • PC ae C38:3 – 1.073 • PC ae C40:2 + 1.353 • PC ae C40:3 – 1.061 • SM (OH) C22:1 + 9.071 • SM(OH) C22:2 + 0.395 • SM(OH) C24:1 – 3.948 • SM C24:1 + 1.832 • SM C26:1 – 2.765 • PC aa C40:4 + 5.387 • PC ae C34:1 – 6.698 • PC ae C36:2 + 4.872 • PC ae C38:2 – 0.717 • PC aa C32:2 – 2.206 • PC aa C36:2 + 4.876 • PC aa C38:4 – 0.256 • PC ae C38:4 – 0.76 • SM C18:1 – 1.541 • SM C18:0 + 0.487 • PC aa C30:2 – 0.863 • PC aa C40:2 + 1.063 • PC aa C42:2 – 0.522 • PC aa C38:6
